# Newborn with Hypoglossia and Micrognathia with Situs Inversus Totalis Born to Mothers with SARS-CoV-2 Infection: A Case Report

**DOI:** 10.3390/diseases13070192

**Published:** 2025-06-24

**Authors:** Gordana M. Velisavljev-Filipovic, Ognjen Jovanov

**Affiliations:** 1 Faculty of Medicine, University of Novi Sad, 21000 Novi Sad, Serbia; ognjen.jovanov.67@gmail.com; 2 Department of Neonatology, Institute for Child and Youth Health Care Vojvodina, 21000 Novi Sad, Serbia

**Keywords:** hypoglossia, micrognathia, situs inversus, newborn, prenatal maternal SARS-CoV-2 infection

## Abstract

Hypoglossia and micrognathia are rare congenital malformations. They are most likely to occur after disruption of blastogenesis during embryonic development and formation of the first pharyngeal arch. They may be associated with other malformations such as otocephaly or hypogenesis syndrome of the oromandibular limb. We present the case of a female infant with hypoglossia, micrognathia, and situs inversus as a very rare triadic combination. This clinical presentation does not correspond to the description of existing syndromes. In the available literature, we were able to find only a small number of described cases that are somewhat similar to ours. The etiology of hypoglossia with micrognathia and situs inversus is unknown and has been attributed to both genetic and teratogenic causes. It is also unclear whether the combination of these three malformations can be classified as its own syndrome or not. Here, we present a child born from a pregnancy exposed to the SARS-CoV-2 virus in the first weeks of embryonic development, whose whole genome sequencing confirmed normality, as a contribution to elucidating the etiology of these congenital malformations. The possible influence of the SARS-CoV-2 virus on the occurrence of these anomalies and the exact mechanism of action should be confirmed in subsequent research.

## 1. Introduction

Hypoplasia of the tongue and mandible is an uncommon combination of congenital malformations, despite their shared origin. The tongue and mandible come from the same embryonic structure. During the fourth week of embryonic development, the first branchial arch divides into maxillary and mandibular processes and tuberculum impar. The latter two form the mandible and the anterior two-thirds of the tongue. The posterior third of the tongue develops from the second and third branchial arches. Hypoglossia and micrognathia most likely develop together, caused by the same unidentified factor, which probably affects the first branchial arch during organogenesis [1]. Disrupted migration or differentiation of neural crest cells can result in conditions such as first arch syndrome. These cells play a crucial role in forming the mesenchyme of the first pharyngeal arch.

Certain genetic conditions are associated with micrognathia and hypoglossia, with Hanhart syndrome and otocephaly being particularly notable examples. While the etiology of Hanhart syndrome is not fully understood, it is hypothesized that vascular abnormalities occurring during embryonic development may play a significant role in its manifestation. Environmental factors during early pregnancy can negatively impact craniofacial development. Conditions such as fetal alcohol syndrome and exposure to retinoic acid, along with various other teratogenic agents, are of concern. Additionally, issues related to abnormal blood flow—such as maternal vascular malperfusion or fetal vascular malperfusion—and vascular events like thrombosis caused by the transmission of the SARS-CoV-2 virus through the placenta can hinder the development of the first pharyngeal arch. Moreover, disruptions in molecular signaling pathways may lead to conditions like hypoglossia and micrognathia.

Isolated hypoglossia cases are rare; more often, it is associated with other congenital disorders, such as limb defects or involving the oromandibular complex (e.g., cleft palate, dental malformations, and maxillomandibular attachment) [2]. Due to potential respiratory difficulties, certain quantitative fetal imaging measurements are often used for clinical decision-making about airway management at birth. Gestational age is important for decision-making [3]. Hypoglossia is classified under the oromandibular-limb hypogenesis syndrome. The exact incidence of hypoglossia in newborns is unknown because the assessment of tongue size itself is subjective and requires careful examination both at rest and during movement. In the last two centuries, fewer than 30 cases of isolated hypoglossia have been reported [2].

Situs inversus totalis, however, is more prevalent in the general population. It is estimated that it occurs in 1 in 8000 to 25,000 people. It can be inherited as an autosomal recessive or X-chromosome-linked trait. However, in a certain number of cases, the existence of this anomaly cannot be found in the family at all. Situs inversus is sometimes associated with heart and/or spleen anomalies, and mucociliary dysfunction [4]. Numerous genetic and environmental factors influence the development of situs abnormalities in humans. Nonetheless, achieving a comprehensive understanding of the cellular and molecular mechanisms that govern asymmetry remains a considerable challenge within this field [5]. There are several reports from hospitals in China describing these malformations. A report from a prenatal care center in China has highlighted a significant increase in the diagnosis of fetal situs inversus among mothers who experienced SARS-CoV-2 infection during early pregnancy, specifically between January and May 2023. This increase was observed following the liberalization of SARS-CoV-2 prevention and control policies, in comparison with mothers who did not have the infection. The findings indicated an odds ratio of 8.2, with a 95% confidence interval ranging from 2.5 to 27.4 [6]. In contrast, a report from Sweden, Denmark, and Norway found no continued increase in the prevalence of situs inversus during the SARS-CoV-2 pandemic in those countries [7].

The triad of micrognathia, hypoglossia (or aglossia), and situs inversus is exceptionally rare in medical literature. It is unclear whether hypoglossia, micrognathia, and situs inversus can stem from the same cause, but we know that this fetus was exposed to SARS-CoV-2 infection during the first 6 weeks of intrauterine, embryonic development.

It is also unclear whether the combination of these three malformations can be classified as its own syndrome or not. Cases like these are sporadic, and information about them is scarce. The presentation of such cases helps add to the knowledge base [8]. This is exactly why it is important to show this case.

## 2. Case Presentation

The patient is a female term newborn who was hospitalized at the Neonatology department of the Pediatric Clinic in Novi Sad, 1 day after birth. The parents are non-consanguineous (an 18-year-old mother and a 19-year-old father) with no history of congenital illnesses or malformations in their respective families. Both individuals are young, healthy, and have recently completed their high school education. The parents’ environment is free from toxic chemicals, heavy metals, and other teratogenic agents. They live in a clean, rural area and were not exposed to harmful influences before conception or during pregnancy. At the beginning of the pregnancy, the mother experienced an elevated temperature of up to 38 °C for four days, accompanied by mild symptoms of fatigue and muscle pain when she was four weeks pregnant. She did not exhibit any respiratory tract symptoms. She did not use any medication other than acetaminophen, which she took during the fourth week of her pregnancy while she was feeling unwell. She experienced symptoms such as diarrhea, vomiting, and headaches. A PCR test for the SARS-CoV-2 virus came back positive, but no further diagnostic testing, whether genetic or otherwise, was conducted until after the birth. In the later weeks of her pregnancy, she felt healthy and showed no symptoms. An ultrasound scan during the 26th week of pregnancy revealed polyhydramnios, but no fetal malformations were detected.

She was delivered at term by Caesarean section, which was indicated due to dystocia. Her birth weight was 3800 g, birth length was 51 cm, and head circumference was 35 cm (all three were 75th centile).

**Figure 1:**
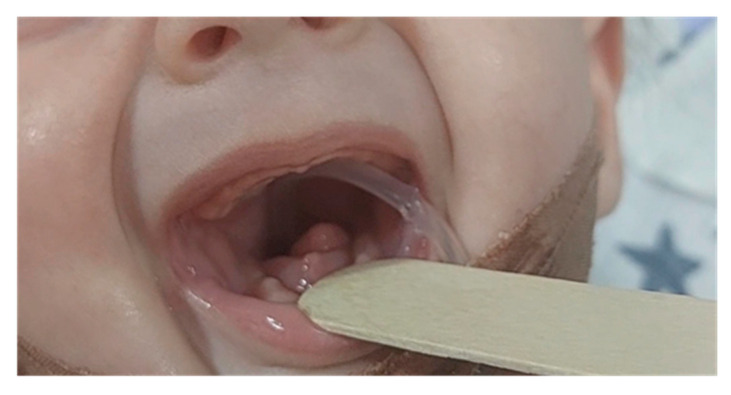
Newborn with hypoglossia and micrognathia without ear anomalies.

Mandibular hypoplasia, hypoglossia, and high-arched palate were evident upon delivery. There was a significant retrognathia, and the anterior two-thirds of the tongue were missing. The posterior third of the tongue was present, and it was bud-shaped and barely motile. This case is depicted in Figure 1. Due to the severity of these malformations, breastfeeding and bottle feeding were not possible. A nasogastric tube was inserted. The newborn was otherwise in good physical condition, and she had no breathing problems. Apgar score was 9 at the 1st, 5th, and 10th minute, respectively, and all vital signs were normal. Physical examination findings also included low-set ears, hypertelorism, broad nasal bridge, and a prolonged and smooth philtrum. Ear canal and tympanic membrane appeared normal on both sides. Hands and feet were normal. There were no other musculoskeletal deformities. During auscultation, heartbeats were mainly heard on the right hemithorax. Chest X-ray and echocardiogram confirmed that there is mirror-image dextrocardia.

**Figure 2:**
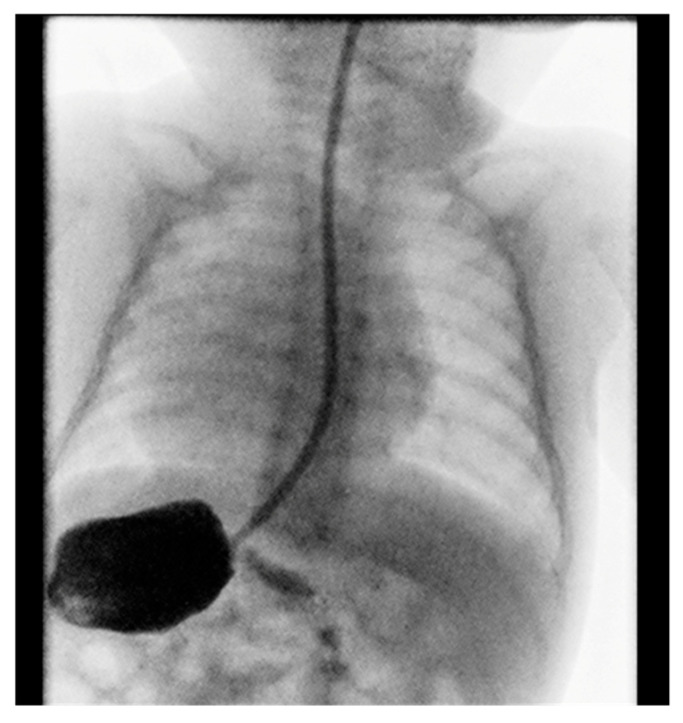
Position of the stomach on the right.

Contrast imaging of the esophagus and stomach shows a normal presentation of the esophagus with the stomach positioned on the right (Figure 2).

Electrocardiogram showed sinus rhythm with right axis deviation. The pulse was 120 beats per minute. Abdominal ultrasound found liver in the left hemiabdomen, and spleen in the right, with all observable visceral organs showing normal morphology and dimensions.

**Figure 3:**
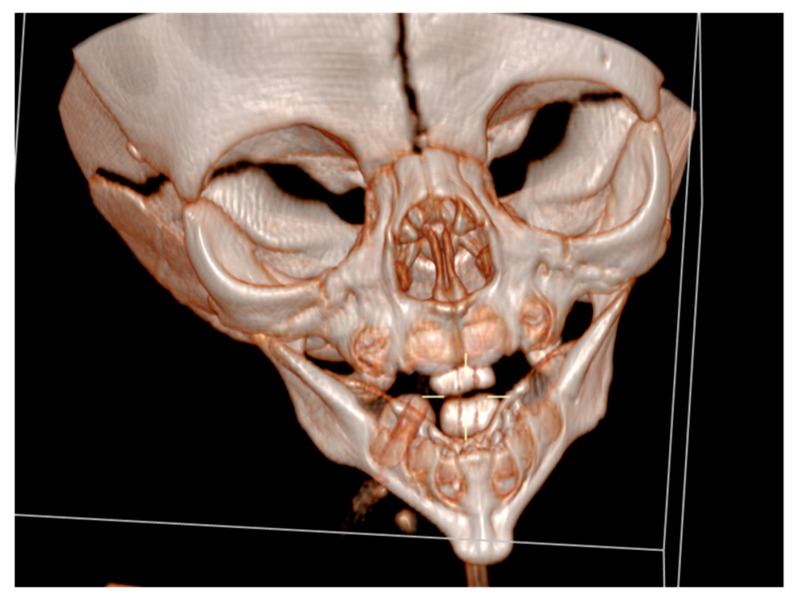
A computed tomography of a narrow and hypoplastic mandibula.

Cranial ultrasound showed no pathological findings, except for discretely hyperechoic periventricular white matter. A computed tomography of the facial region described the mandibula as hypoplastic and narrow (Figure 3).

**Figure 4:**
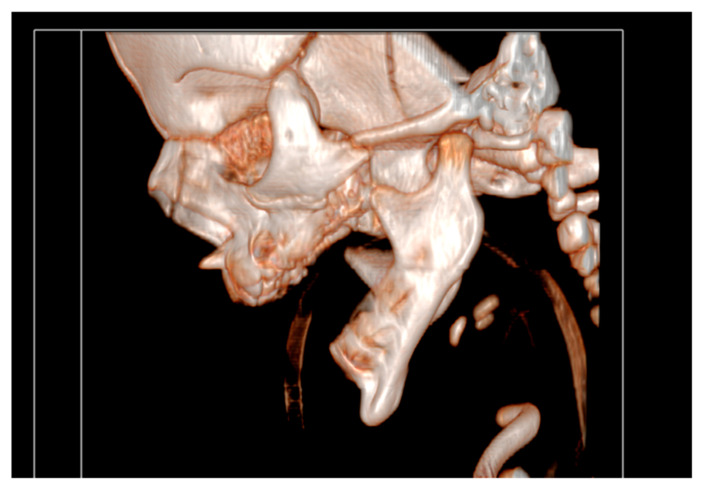
Temporomandibular joints are normal.

It had underdeveloped body region and pointed chin. Only three tooth germs of the lower frontal teeth were present, specifically one lower medial incisor and two lateral incisors. Temporomandibular joints were normal (Figure 4).

**Figure 5:**
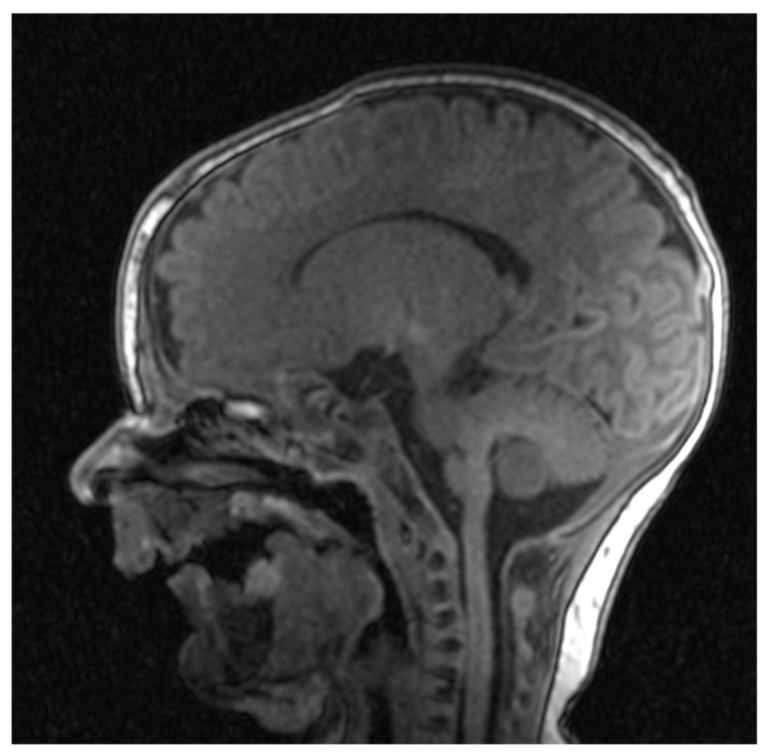
Cranial nuclear magnetic resonance with pronounced micrognathia and oral cavity without a developed tongue.

Cranial nuclear magnetic resonance showed no detectable pathological changes in brain structures and tissues. Brain ventricles and extracerebral cerebrospinal fluid spaces had physiological dimensions. Pronounced mycognathia and oral cavity without a developed tongue (Figure 5).

Pituitary gland and sella turcica were described as normally developed for the age. Ophthalmological examination found no pathological changes in the retina and papilla of the optic nerve of both eyes. Antibodies for TORCH infections, IgG and all IgM, were negative two days after birth.

The otolaryngological findings indicated a rudimentary tongue in the form of a bud with pronounced micrognathia. Examination of the larynx showed normal anatomy without associated anomalies. Hearing screening using transient evoked otoacoustic emission was normal.

Echocardiogram showed a mirror-image dextrocardia. Aorta was shown coming out of anatomical left ventricle, and pulmonary artery was coming out of anatomical right ventricle. Aortic arch was right-positioned, and inferior vena cava could not be clearly visualized. Hepatic veins were found to be flowing directly into the anatomical right atrium. There were no septal defects or shunting. All heart chambers and great vessels had normal dimensions and blood flow.

Gastroduodenal radiography with contrast showed stomach in the right hemiabdomen. Stomach and duodenum had normal structure and motility. There were no signs of hiatus hernia or gastroesophageal reflux, and there were no obstructions to the passage of contrast fluid.

Genetic analysis yielded a normal karyotype, with no microdeletions or microduplications of clinical significance. The genomic analysis and exome sequencing were conducted; however, no pathogenic variants were identified in the results. Newborn primitive reflexes are present and physiological, except for the “rooting” reflex, which is not well coordinated. The absence of the rooting reflex was attributed not to a neurological condition, but rather to an anatomical deficit, specifically microglossia and pronounced micrognathia.

The child notices faces and makes eye contact. For an 8-month-old infant, she showed proper psychomotor development. For a long time, she was fed through an orogastric tube with stimulation of the sucking reflex all the time. At the age of 8 months, milk was swallowed easily, and feeding mushy food became possible, but chewing is still difficult. With intensive speech therapy exercises, the child started to vocalize but does not produce syllables. A recent examination by a physiatrist confirmed that she is capable of sitting independently with good postural alignment and can crawl, although she has not yet achieved the ability to stand. She is under the care of a multidisciplinary team of specialists, including otolaryngologists, orthodontists, ophthalmologists, audiologists, physiatrists, neurologists, and psychologists. The child is progressing well in her psychomotor development. The results of both the electroencephalogram and magnetic resonance imaging of the brain are within normal limits. A comprehensive plan has been developed to address the issue of a narrow mandible and, if necessary, realign the teeth for optimal function. Additionally, speech therapy exercises are currently being implemented to enhance tongue mobility and performance.

## 3. Discussion

Hypoglossia and micrognathia can appear as isolated anomalies, as described by Klaphake et al. [9]. They presented a case of a female patient with hypoglossia, micrognathia, and an 18p deletion on one chromosome. More often, hypoplasia of the tongue and mandible appear together with other congenital anomalies. In the case we described, the associated malformation was situs inversus totalis. This combination of anomalies is extremely rare. We found only a few cases that had a similar clinical presentation. The first described case was reported in the literature in 1925 [10]. Then, in 1971, the case of a 6-year-old girl was reported [11]. Some of them had additional findings, like atrial bradycardia, patent ductus arteriosus, asplenia, polysplenia, and sick sinus rhythm [12,13,14,15].

One of the cases even described that the anterior part of the tongue was present but not fused with the posterior part. These two parts were split by a smooth groove, and the posterior part created breathing problems when the child was in supine position [16].

In the case presented by Chabrolle et al., the pituitary gland was absent [17]. Some of these cases described normal or mildly delayed mental and physical development. Others only confirmed that children were able to live with anomalies at the time of writing [9,11,16,17,18,19,20]. Only two cases ended fatally, due to respiratory distress and asphyxiation, respectively [17,21].

It has been almost 100 years since the first case of hypoglossia, micrognathia, and situs inversus was described in the literature (Watkin, 1925) [10]. There is still no proven cause of these anomalies. Some authors noted that mothers were exposed to fiberglass, nicotine, X-rays, and antibiotics [12,13,14,17,18]. Environmental factors like teratogenic drugs, radiation, hyperthermia, and intrauterine trauma have also been suggested [21]. All exposures happened during the critical period of organogenesis. Still, none of them were proven to have an effect on the development of these anomalies.

In orthodontics, one should always consider etiology. The etiology of malocclusion is often multifactorial, mainly influenced by genetic and environmental factors. In any case, genetic factors or incomplete manifestation of some syndromes that go with malocclusion should be considered [22]. If genetic influence is excluded, environmental factors during fetal development should be considered in detail.

This is exactly why it is important to present our case, in which the mother had fever, symptoms of fatigue, and muscle pain during the first month of pregnancy. She recalls that it was the 4th week of pregnancy, which is a critical period of organogenesis. By testing a nasopharyngeal swab, the PCR test was positive for SARS-CoV-2. We have not found any cases of these anomalies in the literature after the mother’s COVID-19 infection. She only used acetaminophen to treat the fever. This infection is the only plausible cause of deformations that’s left. There is only one other reported case of hypoglossia and situs inversus in which the mother experienced a fever during early pregnancy, and this case was associated with the absence of the pituitary gland in newborns [17]. Some authors suggested that de novo genetic mutations play a role in these malformations, since all parents were healthy with no previous family history of congenital illnesses [23]. Some of the affected children had siblings who were all unaffected and healthy [10,15,20]. One mother had two previous miscarriages of unknown cause [18]. However, children’s karyotypes were all normal, like in our case, except in one case where deletion of one 18p was detected [9].

There have been attempts to classify congenital syndromes that share hypoglossia as a common trait. Such an attempt was made by Hall in 1971 [24]. He proposed a five-type classification of congenital syndromes that have hypoglossia in common. This set of syndromes is known as oromandibular limb hypogenesis syndrome. The spectrum of anomalies that these syndromes include is broad. Most notable are cleft lip and/or palate, micrognathia, hypodactylia, and peromelia. However, they do not include situs inversus.

The malformations present in our patient have only some clinical features close to otocephaly. Otocephaly is classified into four groups: 1. agnathia alone; 2. agnathia with holoprosencephaly; 3. agnathia with situs inversus and visceral anomalies; and 4. agnathia with holoprosencephaly, situs inversus, and visceral anomalies. However, hypoglossia was pronounced in our patient, and holoprosencephaly was not seen.

Otocephaly can also vary in presence and intensity in individual patients. Even if the brain is not affected, death occurs due to pulmonary hypoplasia and severe breathing problems [25]. There has been at least one case where the patient with a mild form of this syndrome lived to the age of 3. Unfortunately, the patient was not observed thereafter [26]. At least four otocephaly cases are known to be caused by a mutation in the gene PRRX1 on the long arm of chromosome 1 [25]. In our patient, genetic analyses did not confirm any pathological mutation.

Given that these three malformations, hypoglossia, micrognathia, and situs inversus, with their phenotypic characteristics and clinical manifestations, do not fully fit into the syndromes described so far, it is suspected that the cases of hypoglossia, micrognathia, and situs inversus in this particular case described could be caused by COVID-19 infection during pregnancy because the course of embryonic development was certainly interrupted at an early stage.

From a pathophysiological perspective, the in utero transmission of SARS-CoV-2 is a viable possibility. In 2021, the World Health Organization (WHO) added SARS-CoV-2 to the list of vertically transmissible infectious agents [27]. Research has demonstrated that the enzymes necessary for the entry of the SARS-CoV-2 virus into cells are present not only within the placenta but also in various tissues of the fetus. The ability of the virus to penetrate the placenta, which functions as a shared organ between the mother and fetus, serves as a conduit to fetal tissues [28]. Consequently, if the virus is able to reach the fetus, there exists a potential for infection to occur. The rate of vertical transmission of SARS-CoV-2 to the fetus necessitates further investigation. By drawing parallels with other pathogens, such as cytomegalovirus and toxoplasmosis, we can better understand the potential for such transmission.

Morhart et al. [29] conducted a study highlighting cases of severe ocular malformations, specifically unilateral microphthalmia, optic nerve hypoplasia, and congenital retinopathy, which were associated with maternal infection by SARS-CoV-2 during the 5th and 6th weeks of embryonic development. Importantly, this embryopathy could not be linked to other infectious agents, genetic factors, drug use, or maternal illnesses experienced during pregnancy. Conversely, it is noteworthy that eight other women who contracted SARS-CoV-2 before the 12th week of gestation delivered healthy infants. The absence of any potentially teratogenic medications taken by the patient’s mother indicates that the infant’s eye abnormalities may be attributed either to materno-fetal transmission of SARS-CoV-2 or to an indirect effect of the maternal SARS-CoV-2 infection during the critical period of optic tract development. However, this conclusion should be approached with caution, as the only link connecting these findings is the fact that the mother contracted COVID-19 during this significant stage of embryogenesis.

The etiology of fetal injury remains inadequately characterized. However, numerous studies indicate that vertical transmission of SARS-CoV-2 from the mother to the developing fetus is a possibility [30].

The SARS-CoV-2 virus has been associated with an increased risk of fetal damage, particularly in cases with more severe clinical presentations. Notably, SARS-CoV-2 placentitis, which is linked to a heightened risk of stillbirth, has also been identified in asymptomatic mothers.

The data currently available suggest that neuropilin-1 (NRP-1), angiotensin-converting enzyme 2 (ACE2), and macrophages play critical roles in the transmission of the virus. Notably, in the context of SARS-CoV-2 transmission, the expression of transmembrane protease serine 2 (TMPRSS2) does not appear to be a significant factor. Both NRP-1 and ACE2 receptors are present on macrophages and monocytes. Furthermore, macrophages that become infected with SARS-CoV-2 are believed to facilitate the virus’s entry into placental and fetal tissues.

The expression of both receptors collectively declines during the later stages of pregnancy, which suggests that the likelihood of vertical transmission is greatest during the first and early second trimesters [31].

The SARS-CoV-2 virus, akin to certain other viruses, possesses the capability to traverse the placental barrier. Research indicates that SARS-CoV-2 can replicate within placental tissue, particularly within syncytiotrophoblasts that are in direct contact with maternal blood, thus allowing for potential infection of the placenta. Furthermore, it is noteworthy that syncytiotrophoblasts lack intercellular gap junctions, enabling pathogens to cross this barrier via leukocytes [32]. The virus may induce inflammation of the placenta, resulting in diminished functionality. This impairment can facilitate the virus’s passage to the fetus without any barriers. Additionally, there is a possibility that the virus causes ischemic damage to the placenta, enabling its transmission to the fetus without directly infecting the placental tissue [33].

It is important to note that if the virus reaches the fetus, infection is possible and could result in embryopathies and congenital anomalies. Additionally, one must consider the physiological immunosuppression experienced by the mother, as well as the limited transfer of anti-SARS-CoV-2 antibodies from the mother to the fetus [34].

Maternal vascular malperfusion (MVM) and fetal vascular malperfusion (FVM) are prevalent histological alterations identified in placentas affected by COVID-19. These changes constitute a significant mechanism through which the COVID-19 virus influences fetal development [33].

COVID-19 infection continues to pose a considerable risk to fetal development [35].

There are several reports from hospitals in China reporting cases of situs inversus associated with SARS-CoV-2 infections. Wang in 2023 describes the association of SARS-CoV-2 Infection during Early Weeks of Gestation with Situs Inversus; A fourfold increase in the frequency of fetal inversus (total inversus situs with dextrocardia or partial situs inversus with levocardia) diagnosed by ultrasound between 20 and 24 weeks of gestation in the period April–June 2023, compared with data from 2014 to 2020 [36].

These studies suggest a potential link between early gestational SARS-CoV-2 infection and disruptions in visceral lateralization, possibly due to direct viral effects or maternal immune responses. Further research is necessary to determine the underlying mechanisms and to rule out other contributing factors.

Recently, a follow-up study to the original report by Wang et al. [36] investigated the timing of SARS-CoV-2 infection and its association with the incidence of fetal situs inversus. Using a matched case–control study design, they estimated an odds ratio of 6.54 (95% CI: 1.76–24.34) for SARS-CoV-2 infection during four to six weeks of gestation, but no increase in infection at other gestational years. This will facilitate further investigation and enhance awareness of the rare complications associated with COVID-19.

A study conducted by Veerus et al. [37] in Estonia indicated that positive SARS-CoV-2 test results during pregnancy were linked to increased rates of stillbirth and neonatal mortality. However, it is important to note that additional research with a larger sample size is necessary to further investigate the long-term health outcomes for children born to mothers who were infected with SARS-CoV-2.

The integration of animal studies and literature findings indicates that transplacental transmission is indeed feasible in the later stages of pregnancy. However, it is important to note that we cannot dismiss the potential for transmission and its associated fetal consequences occurring earlier in the gestational period, as there is currently a lack of definitive data in the existing literature to support this possibility [28].

Although there is currently limited data, it is possible that infections during early pregnancy could interfere with the normal development of the fetus, potentially leading to congenital anomalies. However, additional research is necessary to establish a conclusive relationship between maternal SARS-CoV-2 infection in early pregnancy and embryopathy.

The described rare cases of occurrence of these three anomalies together, for an as-yet-unclear reason, raise the question of whether they represent one entity or not. Given the orderly findings of the examined genome, the question remains open as to which period and at what level the error should occur that leads to the emergence of these three anomalies at the same time.

## 4. Conclusions

Hypoglossia and micrognathia are rare congenital malformations with a wide clinical spectrum. They can be associated with oral and facial malformations as well as anomalies of other organ systems. They most likely occur after the interruption of the normal embryonic development of the tongue and mandible. The causes of its occurrence are numerous, ranging from genetic factors and congenital anomalies to harmful factors from the environment during embryonic development. Here, we present an infant born with hypoglossia, micrognathia, and situs inversus as a very rare triadic combination without additional ear, musculoskeletal, or brain deformities, with normal genome findings. The only thing that has been proven is the exposure of the fetus to the SARS-CoV-2 virus in the first weeks of embryonic development.

This clinical presentation (micrognathia, hypoglossia, situs inversus) does not fit the description of existing syndromes that include similar anomalies, such as otocephaly or oromandibular limb hypogenesis syndrome.

The possible influence of the SARS-CoV-19 virus on the occurrence of these anomalies and the exact mechanism of action should be confirmed in subsequent research.

## Data Availability

The data presented can be provided on request.
